# Enhancing photoelectrochemical performance and stability of Ti-doped hematite photoanode via pentanuclear Co-based MOF modification

**DOI:** 10.3389/fchem.2024.1454524

**Published:** 2024-08-30

**Authors:** Guofa Dong, Fengyan Xie, Fangxia Kou, Tingting Chen, Caihong Xiao, Shaowu Du, Jiaqi Liang, Chenfang Lou, Jiandong Zhuang

**Affiliations:** ^1^ Fuzhou Institute of Oceanography, College of Materials and Chemical Engineering, Minjiang University, Fuzhou, China; ^2^ College of Materials Engineering, Fujian Agriculture and Forestry University, Fuzhou, China

**Keywords:** Co-MOF, polynuclear, Ti doping, hematite, photoelectrochemical water oxidation

## Abstract

Modifying photoanodes with metal-organic frameworks (MOFs) as oxygen evolution reaction (OER) cocatalysts has emerged as a promising approach to enhance the efficiency of photoelectrochemical (PEC) water oxidation. However, designing OER-active MOFs with both high photo- and electrochemical stability remains a challenge, limiting the advancement of this research. Herein, we present a facile method to fabricate a MOF-modified photoanode by directly loading a pentanuclear Co-based MOF (Co-MOF) onto the surface of a Ti-doped hematite photoanode (Ti:Fe_2_O_3_). The resulting Co-MOF/Ti:Fe_2_O_3_ modified photoanode exhibits an enhanced photocurrent density of 1.80 mA∙cm^−2^ at 1.23 V, surpassing those of the Ti:Fe_2_O_3_ (1.53 mA∙cm^−2^) and bare Fe_2_O_3_ (0.59 mA∙cm^−2^) counterparts. Additionally, significant enhancements in charge injection and separation efficiencies, applied bias photon-to-current efficiency (ABPE), incident photon to current conversion efficiency (IPCE), and donor density (N_d_) were observed. Notably, a minimal photocurrent decay of only 5% over 10 h demonstrates the extraordinary stability of the Co-MOF/Ti:Fe_2_O_3_ photoanode. This work highlights the efficacy of polynuclear Co-based MOFs as OER cocatalysts in designing efficient and stable photoanodes for PEC water splitting applications.

## 1 Introduction

Nowadays we are more and more reliable on fossil fuels in our daily life. However, the intensive use of fossil fuel has led to significant environmental issues, including air pollution, global warming, and damage to ecosystems and human health (Hassan et al., 2024). Consequently, there is an urgent need to explore new, less polluting, and more sustainable energy sources. In this context, hydrogen emerges as an ideal candidate to replace fossil fuels due to its clean emission profile, high energy content, sustainability, and diverse applications (Crabtree et al., 2004). Since hydrogen is predominantly available on Earth within water molecules, the most straightforward and environmental method for hydrogen production is to split water molecules into hydrogen and oxygen by electrolysis, particularly using renewable energy sources like solar, hydro, geothermal, and wind (Dincer and Zamfirescu, 2012). Currently, the cost of hydrogen ranges from $2.50 to $6.80 per kilogram. The US Department of Energy (DOE) has set a goal to reduce the cost of hydrogen production to $1 per kilogram by 2030 (Zainal et al., 2024). However, the process of water splitting is energy-intensive, and the sequential conversion of renewable energy into electricity and subsequently into chemical energy (H_2_) can lead to high production costs (Ahad et al., 2023). This economic obstacle is a major technological challenge that must be addressed to facilitate the broader application of hydrogen as a fuel.

Solar-driven photoelectrochemical (PEC) water splitting is an appealing approach to generate hydrogen in a green and cost-competitive way because solar energy is clean, immensely plentiful and inexhaustible (Fujishima and Honda, 1972; Walter et al., 2010). In a typical PEC device, oxygen is produced at the photoanode via the oxygen evolution reaction (OER), while hydrogen is generated at the photocathode through the hydrogen evolution reaction (HER). As OER is a four-electron reaction and is more difficult to occur than HER, research efforts in the improvements of PEC performance have been much focused on the design of photoanode materials. Over recent decades, extensive investigations have been employed on various metal oxide semiconductors, alone or in combination, as potential photoanodes for PEC water splitting (Park et al., 2013; Kment et al., 2017; Zheng et al., 2019; Ma et al., 2020; Liccardo et al., 2022; Zhang et al., 2022). Iron oxide (α-Fe_2_O_3_) is particularly noteworthy, offering several advantageous properties: a suitable band gap (2.1 eV) for the absorption of sunlight, high theoretical solar-to-hydrogen efficiency (15.5%), non-toxicity, affordability, and excellent oxidative stability (Bae et al., 2017; Sivula et al., 2011; Tamirat et al., 2016; Gurudayal et al., 2018; Najaf et al., 2021). Despite of these advantages, however, Fe_2_O_3_ (α is omitted hereinafter) still has many setbacks, including low electrical conductivity and sluggish oxygen-evolution kinetics, which limit its overall PEC efficiency (Zhou and Fan, 2021). To overcome these limitations, various strategies have been employed, such as elemental doping (Zhao et al., 2018; Reddy et al., 2020; Wang et al., 2022; Huang et al., 2024), nanostructure engineering (Kment et al., 2015; Wang et al., 2016; Chnani and Strehle, 2022), heterojunction formation (Bai et al., 2018; Chai et al., 2022; Masoumi et al., 2023), and surface modification (Ahn et al., 2016; Dhandole et al., 2022; Kim et al., 2023). Specifically, surface modifications using oxygen evolution catalysts like cobalt phosphates (Zhong and Gamelin, 2010; Barroso et al., 2011; Carroll et al., 2015), and metal oxides or oxyhydroxides (Morales-Guio et al., 2015; Zhang et al., 2016; Chong et al., 2021) have proven to significantly enhance the PEC performance of Fe_2_O_3_ (Kumar et al., 2022).

In recent years, metal-organic frameworks (MOFs), particularly Co-based MOFs, have been utilized to enhance water oxidation efficiency by directly synthesizing them *in situ* on Fe_2_O_3_ photoanodes (Zhang et al., 2018; Li et al., 2019; Wu et al., 2020; Ali et al., 2021; Wang et al., 2021; Xiao et al., 2021; Cai et al., 2023). The validity of this strategy stems from the advantages of MOFs, such as their large specific surface areas and adjustable pore structures, which allow easy accessibility of catalytic active sites, as well as smooth transport of reactants and products. However, because hydrothermal self-assembly of MOFs is usually not a clean process, fabrication of MOF modified photoanodes through *in situ* synthesis would be encountered with difficulties in purity and loading control of the MOF overlayers. Moreover, the majority of MOFs are not so photo- and electro-chemically stable to be used for practical PEC applications due to their inherent nature of coordination bonds between metal ions and organic ligands. Nevertheless, several researches demonstrated that MOF stability could be effectively improved by replacing single metal nodes with polynuclear cluster nodes (Feng and Du, 2016). In this study, we employ a pentanuclear Co-based MOF, formulated as [(H_3_O)_2_][Co_5_(L)_3_(μ_3_-O)_3_(H_2_O)_3_]·5H_2_O (H_2_L = 2,2-thiodiisonotinic acid), which enhance both the activity and stability of a Ti-doped Fe_2_O_3_ photoanode (Ti:Fe_2_O_3_). This MOF (Co-MOF) features a pentanuclear {Co_5_} cluster node, which is extended into a 3D triangle network through the ligand (L^2−^) (Du et al., 2021). Our results demonstrate that the incorporation of Co-MOF not only improves the PEC activity but most significantly also the overall stability of the modified photoanode Co-MOF/Ti:Fe_2_O_3_.

## 2 Experimental section

### 2.1 Materials

Unless otherwise specified, the reagents used in the experiments were analytical pure and used as received without further purification. Deionized water was used throughout all experiments. Samples of Co-MOF were prepared following the literature method (Du et al., 2021).

### 2.2 Preparation of photoanodes

**FIGURE 1:**
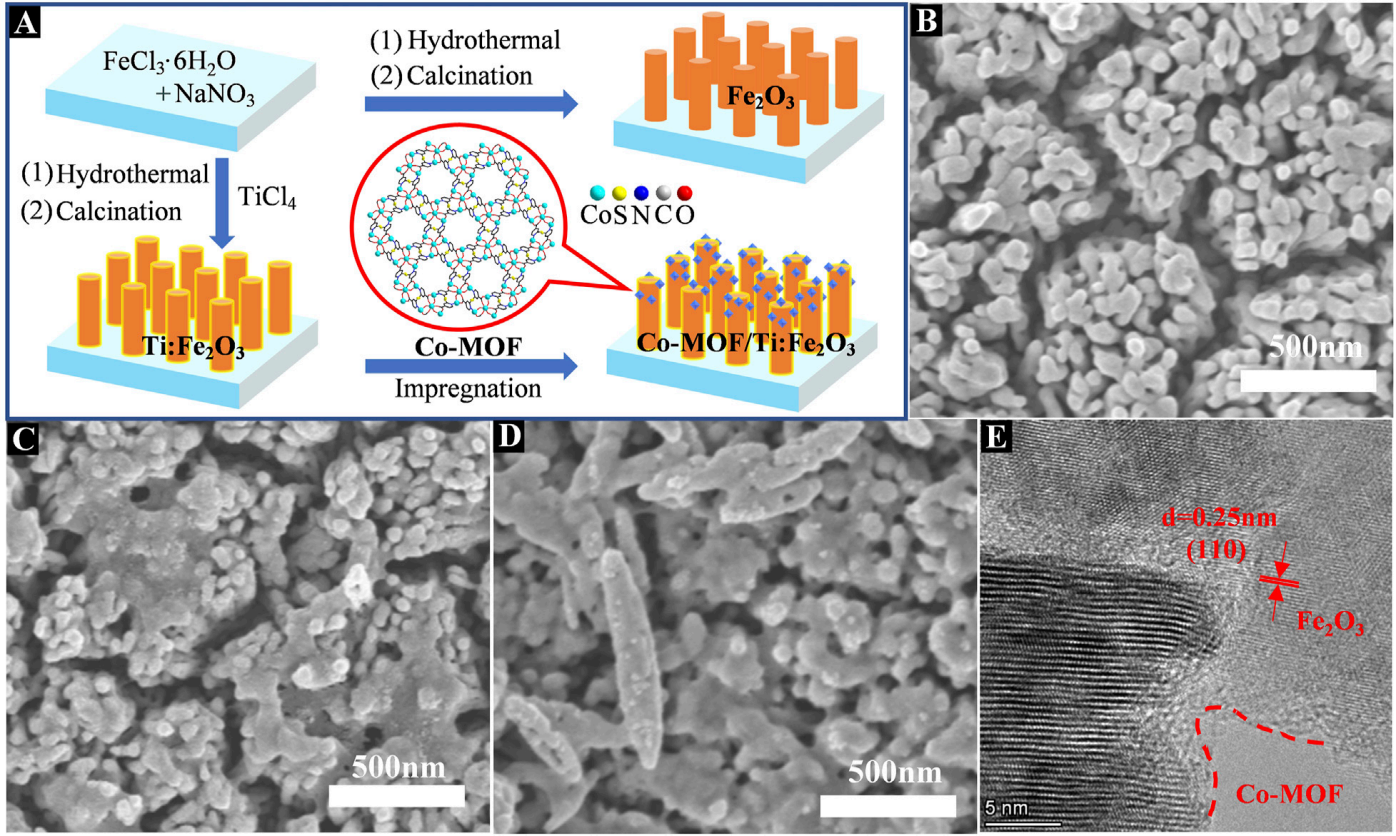
Schematic illustration for the preparation of Co-MOF/Ti:Fe_2_O_3_ photoanode **(A)**; top view SEM images of Fe_2_O_3_ **(B)**, Ti:Fe_2_O_3_ **(C)**, and Co-MOF/Ti:Fe_2_O_3_ **(D)**; HR-TEM image of Co-MOF/Ti:Fe_2_O_3_ **(E)**.

Fe_2_O_3_ materials with or without Ti doping were grown on a fluorine-doped tin oxide (FTO) glass substrates by a modified hydrothermal-annealing method (Bu et al., 2019). Typically, a piece of FTO substrate (30 × 10 × 2.2 mm) was ultrasonically washed with acetone, ethanol and water for 15 min in sequence, followed by drying in an oven at 60°C. The FTO glass substrate was sealed with high-temperature resistant tape, leaving area of 1 × 1 cm^−2^ for following reactions. A 100 mL aqueous solution containing 0.15 M FeCl_3_∙6H_2_O and 1 M NaNO_3_ was stirred for 1 h and a 15 mL of the mixed solution was transferred to a 25 mL Teflon-lined stainless steel autoclave. A clean FTO glass was immersed into the solution and the autoclave was heated at 95°C for 4 h. The yellow film obtained was washed repeatedly with water and annealed in muffle oven at 550°C (5°C/min) for 2 h and subsequently at 770°C (10 °C/min) for 15 min in air to obtain a Fe_2_O_3_ film. The preparation of Ti-doped Fe_2_O_3_ film was exactly the same as above, except that 1 μL TiCl_4_ was added to the solution before it was transferred to the autoclave. The as-prepared Fe_2_O_3_ and Ti-doped Fe_2_O_3_ photoanodes are designated as Fe_2_O_3_ and Ti:Fe_2_O_3_, respectively. For Co-MOF modified photoanode, a sample of Co-MOF (2 mg) was dispersed in 1 mL of ethanol and Nafion solution (v/v: 1:100) through sonication. The Ti:Fe_2_O_3_ photoanode was immersed in the above suspension for 5 min, then dried at 60°C for 5 min in oven. The above process was repeated once to obtain the Co-MOF/Ti:Fe_2_O_3_ composite photoanode (Figure 1A).

### 2.3 Material characterization

The surface morphology of the samples was examined using a Hitachi SU8000 scanning electron microscope (SEM) and a JEOL 2100 High-resolution transmission electron microscope (HRTEM). The element distribution was analyzed by energy-dispersive X-ray spectroscopy (EDS) and mapping. The X-ray diffraction (XRD) measurements were performed on a Mini FLEX600 spectrometer with Cu-Kα radiation. X-ray photoelectron spectroscopy (XPS) measurements were carried out using an EscaLab 250Xi (Thermo Fisher Scientific, USA) with an achromatic Al Kα source (1486.6 eV). No surface cleaning was employed before the XPS analysis (Bae et al., 2020). Photoluminescence (PL) spectra were taken by using an Edinburgh FLS1000 fluorescence spectrometer.

### 2.4 Photoelectrochemical measurements

The PEC performances were measured by an electrochemical workstation (CHI 660E) using a standard three-electrode configuration with as-prepared photoanodes as the working electrode, a platinum foil (1 × 1 cm) as the counter electrode, a saturated Ag/AgCl electrode as the reference electrode and 1.0 M NaOH (pH = 13.4) as the electrolyte. A 300 W xenon lamp (PLS-FX300HU) coupled with an AM 1.5 G filter was used as the light source, and the light intensity was adjusted to 100 mW∙cm^2^. All as-prepared photoanodes were illuminated from the back side and the irradiated areas were 1.0 cm^2^. All electrode potentials reported herein were converted to the reversible hydrogen electrode (RHE) using equation E vs RHE = E (Ag/AgCl) + 0.197 + 0.059 × pH. The linear sweep voltammogram (LSV) curves were recorded at a scan rate of 5 mV/s from −0.4–0.6 V. The photoelectrochemical impedance spectroscopy (PEIS) was performed under illumination with a frequency ranging from 0.01 to 100 kHz and the perturbation amplitude is 5 mV. The Motte-Schottky (M-S) plots were evaluated in dark at a frequency of 1 kHz with a perturbation amplitude of 5 mV. Durability test of the photoanodes was conducted under successive illumination for 10 h at 1.23 V. The evolved H_2_ and O_2_ were collected and tested in a three-electrode system by a gas chromatograph spectrometer (GC9790Ⅱ) with a thermal conductivity detector (TCD). The electrolyte was purged with Ar for 30 min to eliminate any dissolved oxygen before the measurement.

## 3 Results and discussion

### 3.1 Characterization of the photoanodes

The morphology, crystal structure, and elemental composition of the samples were characterized using scanning electron microscopy (SEM) and transmission electron microscopy (TEM). As depicted in Figure 1B and Supplementary Figure S1D, Fe_2_O_3_ nanorods, with mean diameters ranging from 70 nm to 100 nm, are uniformly grown perpendicular to the FTO substrate. After Ti doping, the morphology of Ti:Fe_2_O_3_ remains largely unchanged, though slight necking and coalescence among adjacent nanorods are observed (Figure 1C). The SEM images show that after loading, Co-MOF particles are evenly dispersed on the surface of the Ti:Fe_2_O_3_ nanorod layer (Figure 1D). Corresponding TEM analyses confirm the one-dimensional rod-like morphology of both Fe_2_O_3_ and Ti:Fe_2_O_3_, along with a homogeneous distribution of Co-MOF particles (Supplementary Figure S1). HR-TEM images of Co-MOF/Ti:Fe_2_O_3_ display a lattice spacing of 0.25 nm, which corresponds to the (110) plane of Fe_2_O_3_ (Figure 1E) (Zhang et al., 2019). The elemental composition was further analyzed using EDX (Supplementary Figure S2 and Table S1) and elemental mapping via SEM (Supplementary Figure S3), confirming a uniform distribution of Fe, Co, Ti, O, N, and S over the sample.

**FIGURE 2:**
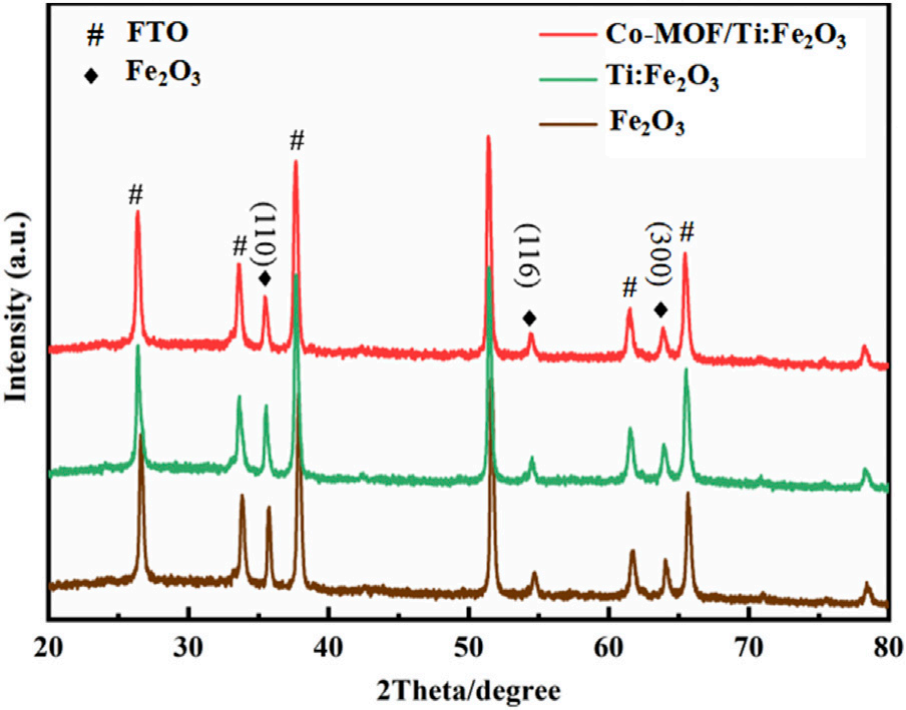
XRD patterns of the photoanodes.

The crystalline structures of Fe_2_O_3_, Ti:Fe_2_O_3_, and Co-MOF/Ti:Fe_2_O_3_ were characterized using XRD. As depicted in Figure 2, all photoanode materials exhibit diffraction peaks at 35.61°, 54.09°, and 63.99°, corresponding to the (110), (116), and (300) planes of Fe_2_O_3_ (JCPDS: 33-0664), respectively (Wang et al., 2018a). Additional peaks are indexed to SnO_2_ (JCPDS: 46-1088), originating from the FTO substrate (Liu et al., 2019). The XRD profile for Ti:Fe_2_O_3_ is similar to that of pure Fe_2_O_3_, confirming the successful incorporation of Ti without the formation of new crystalline phases. No diffraction peaks were observed for the Co-MOF, likely due to its minimal content level.

To elucidate the surface chemical composition and electronic states of the photoanodes, XPS analyses were conducted (Supplementary Figure S4, S5). The comprehensive XPS survey confirmed the presence of Fe, O, Ti, Co, and S in Co-MOF/Ti:Fe_2_O_3_. In the fine Fe 2p spectrum of Co-MOF/Ti:Fe_2_O_3_, three distinct peaks were observed: Fe 2p_3/2_ at 711.8 eV, Fe 2p_1/2_ at 724.5 eV, and a satellite peak at 719.1 eV, corroborating the presence of Fe₂O₃ (Peng et al., 2021). The O 1s spectrum of Co-MOF/Ti:Fe_2_O_3_ was deconvoluted into three distinct peaks: lattice oxygen at 530.5 eV, surface hydroxyl groups at 532.6 eV, and water molecules at 535.5 eV (Wei et al., 2015). Compared to Fe_2_O_3_ and Ti:Fe_2_O_3_, the binding energies of Fe 2p and O 1s in Co-MOF/Ti:Fe₂O₃ exhibited positive shifts of approximately 0.6 and 0.7 eV, respectively. These shifts suggest potential electronic couplings at the interface between Co-MOF and Ti:Fe₂O₃, likely enhancing photogenerated charge transfer (Li et al., 2020). Additionally, a new area at 532.6 eV was noted, attributable to the oxygen species from the organic ligands in Co-MOF. The Ti 2p XPS spectra of Ti:Fe_2_O_3_ and Co-MOF/Ti:Fe_2_O_3_ revealed two symmetric peaks at around 458.2 eV and 464.0 eV, with a splitting energy of ∼5.6 eV, indicative of Ti doping (Peng et al. 2021). The Co 2p spectrum of Co-MOF/Ti:Fe_2_O_3_ displayed peaks at 781.1 eV and 795.7 eV, corresponding to Co 2p_3/2_ and Co 2p_1/2_, respectively, confirming the presence of Co^2^⁺ (Du et al., 2021).

### 3.2 Photoelectrochemical properties

**FIGURE 3:**
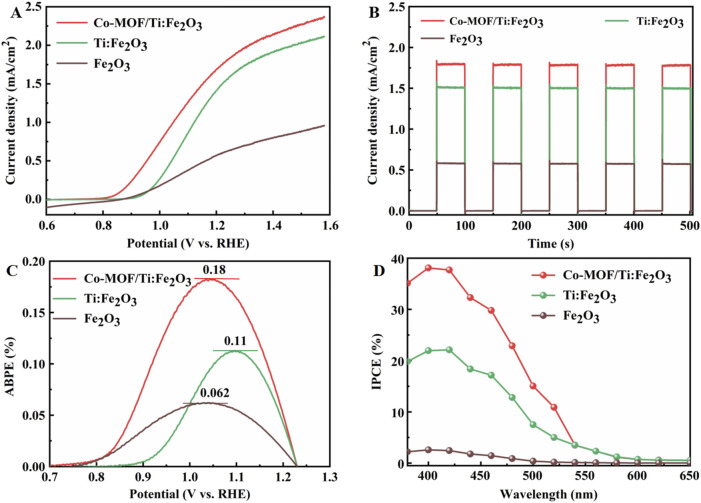
LSV curves at 5 mV/s under AM 1.5 G illumination **(A)**, chopped I-t curves at 1.23 V **(B)**, ABPE **(C)** and IPCE **(D)** spectra of the photoanodes.

The linear sweep voltammetry (LSV) curves of the photoanodes were recorded under AM 1.5 G illumination. Fe_2_O_3_ and Ti:Fe_2_O_3_ exhibited photocurrent densities of 0.59 mA·cm^−2^ and 1.53 mA·cm^−2^ at 1.23 V, respectively. In contrast, the Co-MOF/Ti:Fe_2_O_3_ photoanode demonstrated a superior photocurrent density of 1.80 mA·cm^−2^ (Figure 3A). Additionally, Co-MOF/Ti:Fe_2_O_3_ displayed an onset potential of only 0.86 V, showing a 100 mV cathodic shift relative to Ti:Fe_2_O_3_. The chopped current-time (I−t) curves for these photoanodes, measured at 1.23 V under interrupted illumination, are presented in Figure 3B. All samples showed rapid photocurrent response and achieved a stable current upon light activation, indicating excellent light sensitivity and robust stability. The photocurrent densities are consistent with those observed in the LSV results. The PEC performance of Co-MOF/Ti:Fe_2_O_3_ is either better than or comparable to previously reported results (Supplementary Table S2). This enhancement is attributed to the improved conductivity from Ti doping and the enhanced surface oxidation kinetics due to Co-MOF, synergistically advancing the PEC efficiency of the hematite photoanode.

The ABPE of the photoanodes are detailed in Figure 3C. Co-MOF/Ti:Fe_2_O_3_ achieved a ABPE value of 0.18% at 1.05 V, which is significantly higher than that of Fe_2_O_3_ (0.062% at 1.03 V) and Ti:Fe_2_O_3_ (0.11% at 1.09 V), representing increases of 2.9 times and 1.6 times, respectively. Furthermore, the IPCE were measured, with results shown in Figure 3D. All photoanodes demonstrated photoresponses across the wavelength range of 380 nm–650 nm, with IPCE values at 400 nm being 2.6% for Fe_2_O_3_, 20.9% for Ti:Fe_2_O_3_, and 38.1% for Co-MOF/Ti:Fe_2_O_3_. The enhanced ABPE and IPCE for Co-MOF/Ti:Fe_2_O_3_ can be attributed to improved charge transfer kinetics on the surface facilitated by the incorporation of Co-MOF.

**FIGURE 4:**
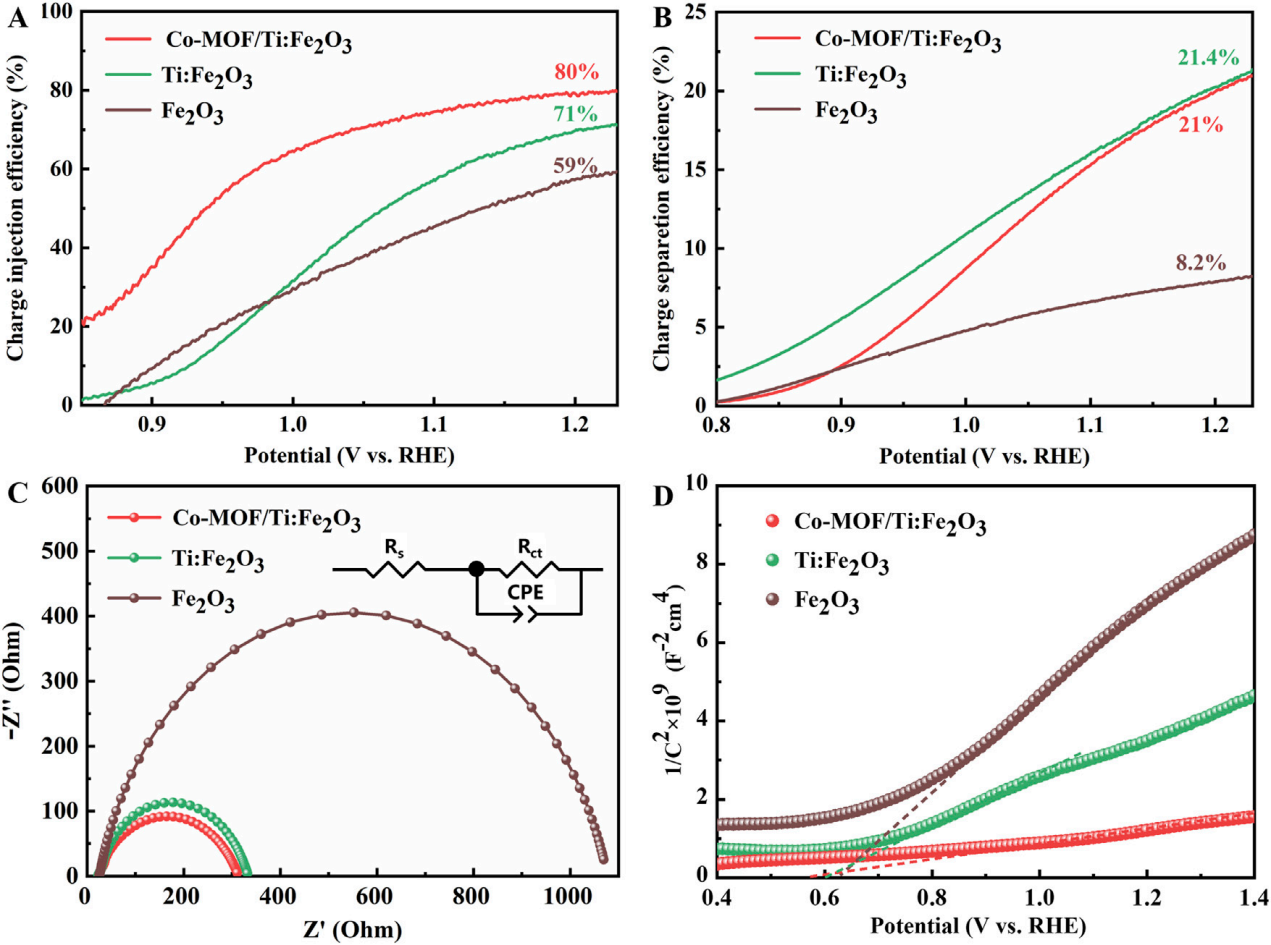
Charge injection efficiency **(A)**, charge separation efficiency **(B)**, Nyquist plots **(C)**, and Mott–Schottky plots **(D)** of the photoanodes. Inset in **(C)** is the equivalent electric circuit for impedance fitting.

To clarify the roles of Ti doping and the Co-MOF co-catalyst, the photocurrent densities of photoanodes were evaluated under illumination from an AM 1.5 G light source in a 1 M NaOH electrolyte, both with and without a 0.5 M Na_2_SO_3_ hole sacrificial agent, as depicted in Supplementary Figure S6. The efficiencies of charge injection and separation for these photoanodes are presented in Figures 4A, B respectively. The charge injection efficiency for the Co-MOF/Ti:Fe_2_O_3_ photoanode reached 80%, superior to that of Ti:Fe_2_O_3_ (71%) and bare Fe_2_O_3_ photoanode (59%). The charge separation efficiency for the Co-MOF/Ti:Fe_2_O_3_ (21.0%) was comparable with that of Ti:Fe_2_O_3_ (21.4%), both of which were much higher than that of bare Fe_2_O_3_ photoanode (8.2%). These results demonstrat significant improvements in reducing surface recombination and enhancing water oxidation kinetics through the synergistic effects of Ti doping and Co-MOF loading.

Photoelectrochemical impedance spectroscopy (PEIS) measurements provide insights into the charge transfer behavior of photoanodes under illumination. Figure 4C displays the Nyquist plots for the bare Fe_2_O_3_, Ti:Fe_2_O_3_, and Co-MOF/Ti:Fe_2_O_3_ photoanodes, which were analyzed using an equivalent resistance-capacitance (RC) circuit model (inset in Figure 4C). Within this model, R_s_ represents the series resistance associated with the FTO substrate and the test system, while R_ct_ denotes the charge transfer resistance at the electrode-electrolyte interface during the water oxidation reaction. The fitted parameters for these resistances for the different photoanodes are detailed in Supplementary Table S3. Notably, all photoanodes display similar external resistances (R_s_) ranging from 23.1 Ω to 25.2 Ω, indicating a consistent interface between the semiconductor and the FTO substrate. However, the Co-MOF/Ti:Fe_2_O_3_ photoanode demonstrates a significantly lower R_ct_ value of 289 Ω compared to Fe_2_O_3_ (1,047 Ω) and Ti:Fe_2_O_3_ (310 Ω). These results suggest that Ti doping and the addition of Co-MOF on Fe_2_O_3_ can markedly enhance the charge transfer processes at the electrode/electrolyte interface, contributing to improved PEC performance.

Mott-Schottky (M-S) analysis was utilized to further assess the donor concentration (N_d_) and the flat band potential (V_fb_) of the photoanodes. The positive slopes of the M-S plots confirm the n-type semiconductor characteristics of these hematite photoanodes. A decrease in the slope indicates an increase in carrier density, reflecting enhanced charge carrier concentration in the modified hematite photoanodes (Figure 4D). The N_d_ value for the Co-MOF/Ti:Fe_2_O_3_ photoanode was determined to be approximately 9.32 × 10^20^ cm^−3^. In comparison, the N_d_ values for the Ti:Fe_2_O_3_ and bare Fe_2_O_3_ photoanodes were 2.63 × 10^20^ cm^−3^ and 1.42 × 10^20^ cm^−3^, respectively (Supplementary Table S4). This result suggests that Ti doping and Co-MOF loading incrementally enhance the charge transfer capabilities of the photoanodes (Kumar et al., 2022). The flat band potentials (V_fb_) were measured as 0.56 V for Co-MOF/Ti:Fe_2_O_3_, 0.61 V for Ti:Fe_2_O_3_, and 0.63 V for bare Fe_2_O_3_, which is consistent with the trend of the onset potentials observed in the LSV curves.

The steady-state open-circuit photovoltage (OCP) was measured both under light illumination and in the dark, as shown in Supplementary Figure S7. For Fe_2_O_3_, the OCP in the dark is recorded at 0.905 V, deviating from the equilibrium value of 1.23 V and indicating the presence of surface states. After Ti doping and Co-MOF loading, the OCP in the dark shifts positively (0.97 V for Ti:Fe_2_O_3_ and 1.02 V for Co-MOF/Ti:Fe_2_O_3_), suggesting partial passivation of these surface states, and hence the enhancement of electron-hole separation and surface kinetics. A higher photovoltage of Co-MOF/Ti:Fe_2_O_3_ photoanode also indicates facile hole transfer from Ti:Fe_2_O_3_ to Co-MOF with abundant active sites, promoting the PEC water oxidation. Surprisingly, the open-circuit potential difference (V_oc_ = V_dark_ − V_light_) decreases from 0.16 V for Fe_2_O_3_ to 0.12 V for Ti:Fe_2_O_3_ and further to 0.13 V for Co-MOF/Ti:Fe_2_O_3_. This reduction in photovoltage for the Ti-doped samples could be due to the increased carrier density, which narrows the space charge layer, a phenomenon also observed in other similar systems (Cai et al., 2023). Photoluminescence (PL) measurements were conducted in air without applied bias to assess the impact of Ti doping and Co-MOF loading on charge separation, as illustrated in Supplementary Figure S8. A notable reduction in PL intensity for Ti:Fe_2_O_3_ compared to bare Fe_2_O_3_ corresponds with the decreased charge carrier recombination and enhanced electron and hole separation efficiency (Wang et al., 2018b). Conversely, the Co-MOF modification led to an increase in PL intensity, which suggests effective hole storage within the material, enhancing the likelihood of hole transfer to the electrode surface during PEC water oxidation (Zhang et al., 2018; Wu et al., 2020).

**FIGURE 5:**
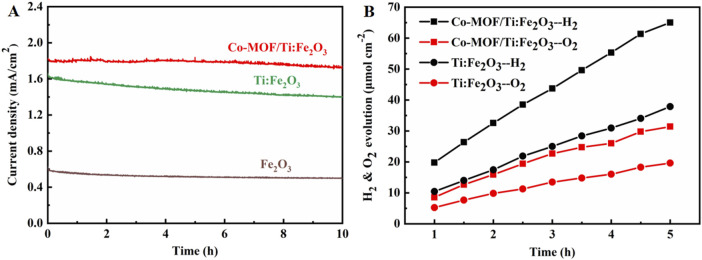
**(A)** Chronoamperometry of the photoanodes in a period of 10 h at 1.23 V under simulated AM 1.5 G illumination; **(B)** evolution of H_2_ and O_2_ gases from Ti:Fe_2_O_3_ and Co-MOF/Ti:Fe_2_O_3_ photoanodes at 1.23 V.

Stability of the photoanodes was examined through chronoamperometry (Figure 5A). The Co-MOF/Ti:Fe_2_O_3_ photoanode demonstrated excellent stability, maintaining 95% of its initial photocurrent density throughout the test duration. In contrast, the bare Fe_2_O_3_ and Ti:Fe_2_O_3_ photoanodes retained only 80% and 84% of their original activity, respectively. Subsequently, the generation and quantification of H_2_ and O_2_ by the Co-MOF/Ti:Fe_2_O_3_ photoanode were conducted and are presented in Figure 5B. The Co-MOF/Ti:Fe_2_O_3_ photoanode achieved PEC H_2_ and O_2_ evolution rates of 13.0 μmol·cm^−2^·h^−1^ and 6.28 μmol·cm^−2^·h^−1^, respectively. In comparison, the Ti:Fe_2_O_3_ photoanode exhibited lower evolution rates of 7.58 μmol·cm^−2^·h^−1^ for H_2_ and 3.92 μmol·cm^−2^·h^−1^ for O_2_. This results indicate the superior PEC stability of the Co-MOF/Ti:Fe_2_O_3_ photoanode, highlighting its effectiveness in sustained water splitting under operational conditions.

### 3.3 Conclusions

In conclusion, the performance of a hematite photoanode in PEC water splitting was enhanced through Ti doping and Co-MOF modification. The modified photoanode, Co-MOF/Ti:Fe_2_O_3_, was easily fabricated by directly loading Co-MOF particles onto the surface of the Ti-doped hematite photoanode. LSV measurements under illumination demonstrated that modifying with Co-MOF increased the photocurrent density at 1.23 V from 1.53 mA∙cm^−2^ for Ti:Fe_2_O_3_ and 0.59 mA∙cm^−2^ for Fe_2_O_3_ to 1.80 mA∙cm^−2^, while shifting the onset potential by 100 mV compared to Ti:Fe_2_O_3_. Additionally, the modification led to reduced charge transfer resistance at the electrode-electrolyte interface, as well as enhanced charge injection and separation efficiencies, ABPE and IPCE values, and donor density. The improved PEC performance of Co-MOF/Ti:Fe_2_O_3_ can be attributed to the Ti doping and loading of Co-MOF co-catalyst, both of which reduce surface charge recombination and improve charge transfer and water oxidation kinetics. Furthermore, the polynuclear cluster nodes in Co-MOF enhance framework connectivity, ensuring MOF integrity during the PEC process. Consequently, the Co-MOF modified photoanode exhibited remarkable stability, with only a 5% decrease in photocurrent after 10 h of PEC, which is superior to most of the recently reported MOF-modified hematite photoanodes. This study provides valuable insights for the development of stable MOF materials for PEC water splitting applications.

## Data Availability

The original contributions presented in the study are included in the article/Supplementary Material, further inquiries can be directed to the corresponding author.
