# Supplementary material for: Enhancing photoelectrochemical performance and stability of Ti-doped hematite photoanode via pentanuclear Co-based MOF modification
Source: Front Chem. 2024 Aug 30;12:1454524. doi: 10.3389/fchem.2024.1454524 (PMC11392840; doi:10.3389/fchem.2024.1454524)
Supplement: Supplementary file 1 [file DataSheet1.docx]

Supplementary Material

**Enhancing Photoelectrochemical Performance and Stability of Ti-Doped Hematite Photoanode via Pentanuclear Co-based MOF Modification**

**Guofa Dong^1^, Fengyan Xie^1^, Fangxia Kou^1^, Tingting Chen^1^, Caihong Xiao^1^, Shaowu Du^1^*, Jiaqi Liang^2^, Chenfang Lou^2^ and Jiandong Zhuang^2^**

^1^ *Fuzhou Institute of Oceanography, College of Materials and Chemical Engineering, Minjiang University, Fuzhou 350108, China*

^2^ *College of Materials Engineering, Fujian Agriculture and Forestry University, Fuzhou 350002, China*

* Correspondence:
Corresponding Author: Shaowu Du
email: [swdu@mju.edu.cn](mailto:swdu@mju.edu.cn)


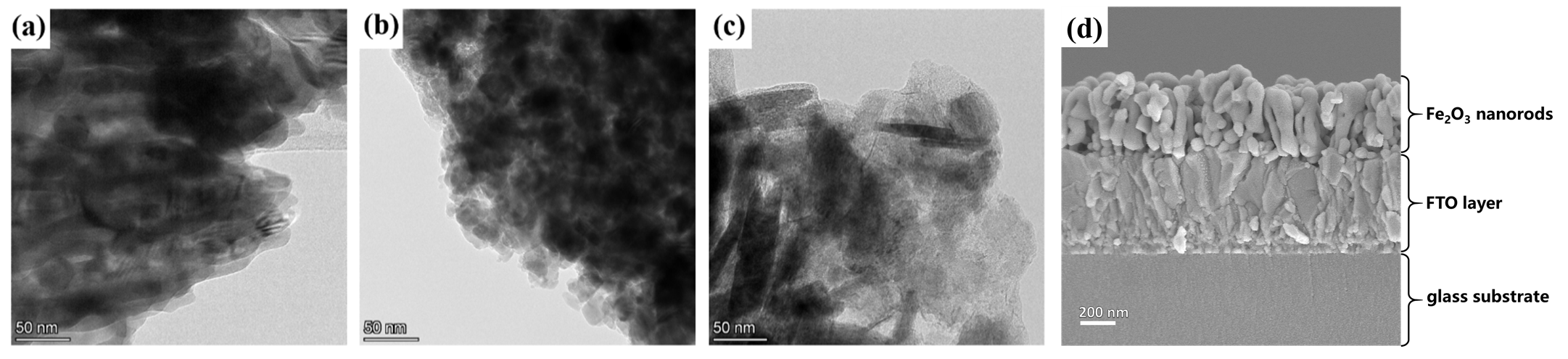


**Figure S1.** TEM images of (a) Fe_2_O_3_; (b) Ti:Fe_2_O_3_; (c) Co-MOF/Ti:Fe_2_O_3_, and the cross-section SEM image of the Fe_2_O_3_ photoelectrode (d).


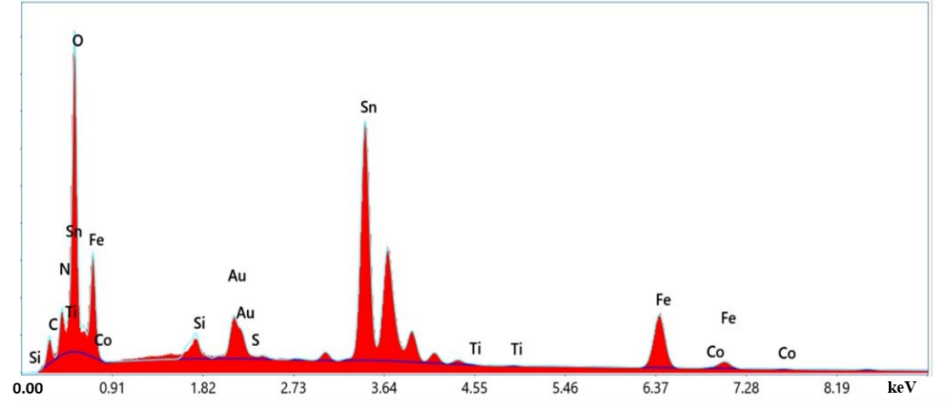


**Figure S2.** Energy-dispersive X-ray spectroscopy spectrum of Co-MOF/Ti:Fe_2_O_3_ photoanode.


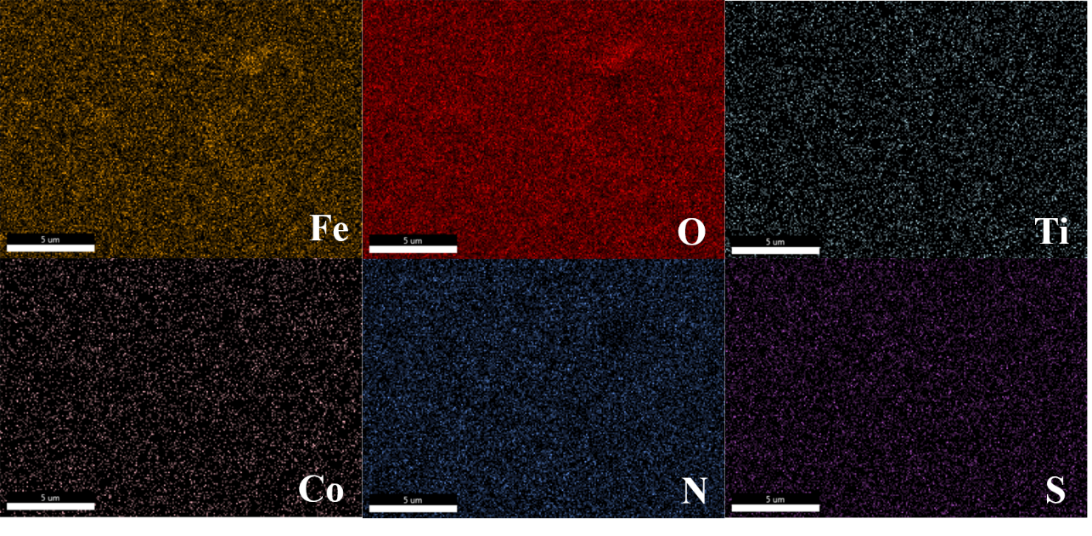


**Figure S3.** Elemental mappings images of Co-MOF/Ti:Fe_2_O_3_ photoanodes.





**Figure S4.** The comprehensive XPS spectra of the photoanodes.


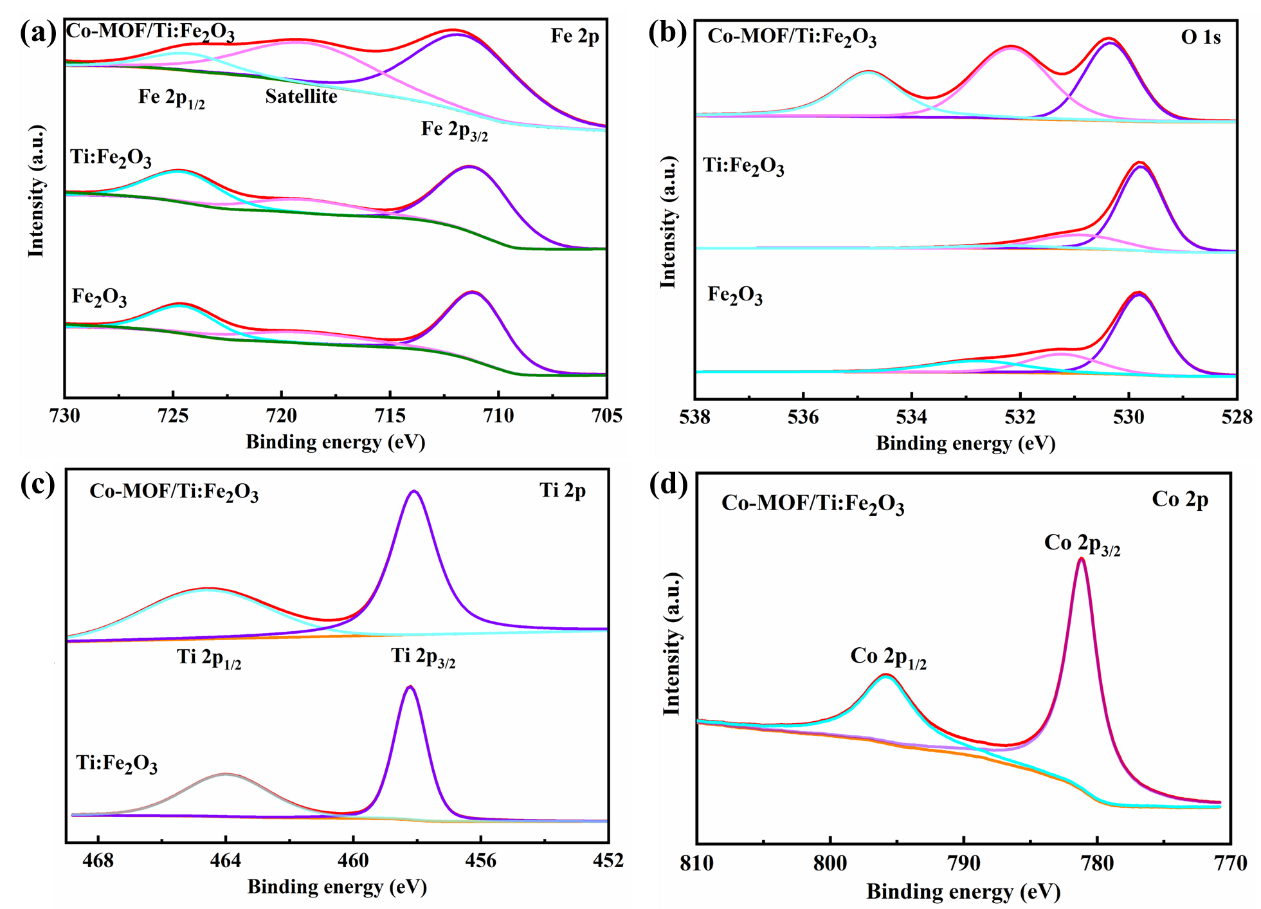


**Figure S5.** High-resolution XPS spectra of (a) Fe 2p, (b) O 1s, (c) Ti 2p, and (d) Co 2p for the photoanodes.





**Figure S6.** LSV curves of the photoanodes in a 1 M NaOH electrolyte with (dash line) and without (solid line) 0.5 M Na_2_SO_3_.





**Figure S7.** OCP of the photoanodes under darkness and illumination.


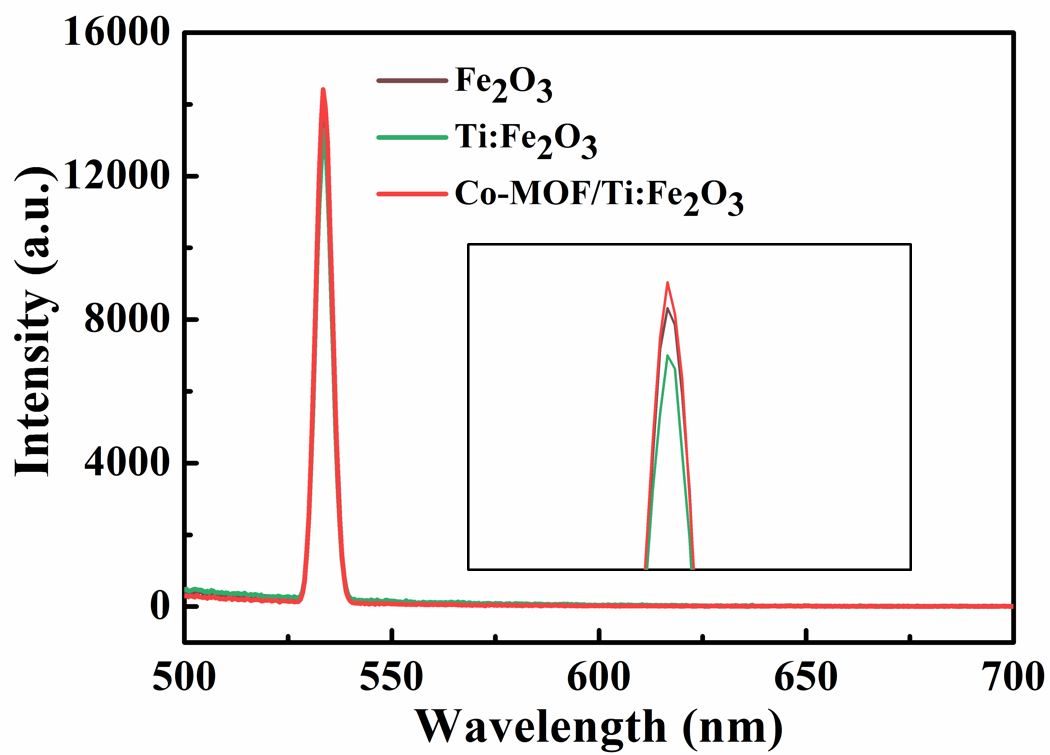


**Figure S8.** PL emission spectra of the photoanodes under 365 nm excitation. Inset presents enlarged of peak maxima.

**Table S1.** Elemental analysis for Co-MOF/Ti:Fe_2_O_3_ photoanode.

| **Elements** | **Weight %** | **Atomic %** |
| --- | --- | --- |
| C K | 1.7 | 5.6 |
| N K | 2.0 | 5.6 |
| O K | 22.5 | 56.0 |
| Si K | 1.2 | 1.7 |
| S K | 0.2 | 0.2 |
| Ti K | 0.2 | 0.2 |
| Fe K | 18.6 | 13.3 |
| Co K | 0.5 | 0.3 |
| Sn L | 48.1 | 16.1 |
| Au M | 5.1 | 1.0 |

**Table S2.** The recent literature summary of Fe_2_O_3_-based photoanodes at 1.23 V vs. RHE under AM 1.5 G illumination (100 mW cm^−2^).

| Photoanodes | J_ph_  (mAcm^−2^) | η_inject_  (%) | IPCE  (%) | ABPE  (%) | Stability  (hour) | Refs |
| --- | --- | --- | --- | --- | --- | --- |
| FeCo-MOF/Fe_2_O_3_ | 2.80 | 95.0 | 79.0 | 0.25 | 5.0 | 1 |
| MIL-101/Fe_2_O_3_ | 1.00 | / | 25.0 | 0.13 | 5.0 | 2 |
| Sn-Ti-Fe_2_O_3_/ZIF-67 | 2.00 | 68.6 | / | 0.17 | / | 3 |
| Fe_2_O_3_:Ti/NH_2_-MIL-101(Fe) | 2.27 | 69.3 | 42.3 | / | 0.7 | 4 |
| Fe_2_O_3_@0.1Co@0.8MIm | 2.00 | / | 13.1 | / | / | 5 |
| Fe@Ni–MOF/Fe_2_O_3_:Ti | 2.30 | 89.0 | 34.2 | 0.15 | 2.0 | 6 |
| Co-MOF/Ti-Fe_2_O_3_ | 1.01 | 88.0 | / | 0.09 | / | 7 |
| Co-MOF@TiFe | 1.24 | 88.8 | / | / | 0.1 | 8 |
| Ni-MOF/ Fe_2_O_3_ | 0.85 | 58.0 | 9.0 | / | 2.0 | 9 |
| Co-MOF/Ti-Fe_2_O_3_ | 2.24 | 80.4 | / | 0.17 | 6.0 | 10 |
| Ti:Fe_2_O_3_@MOF | 2.10 | / | 12 | 0.25 | / | 11 |
| Fe_2_O_3_@ZIF-67 | 0.85 | 85 | 23.3 | 0.07 | 1.9 | 12 |
| Ti-Fe_2_O_3_/CoFePi | 1.75 | 75.8 | 46.6 | 0.15 | 3.0 | 13 |
| Ti-Fe_2_O_3_/Pi | 1.56 | 77.6 | 27.5 | 0.12 | 2.8 | 14 |
| Co-MOF/Ti:Fe_2_O_3_ | 1.80 | 80.0 | 38.1 | 0.18 | 10.0 | This work |

**Table S3.** EIS results of the photoanodes.

| Samples | R_s_(Ω) | R_ct_(Ω) |
| --- | --- | --- |
| Fe_2_O_3_ | 25.2 | 1047 |
| Ti:Fe_2_O_3_ | 23.3 | 310 |
| Co-MOF/Ti:Fe_2_O_3_ | 23.1 | 289 |

**Table S4.** Flat band potentials, slope, and carrier density of the photoanodes.

| Samples | V_fb_(V) | Slope | N_d_(cm^−3^) |
| --- | --- | --- | --- |
| Fe_2_O_3_ | 0.63 | 1.242×10^10^ | 1.42×10^20^ |
| Ti:Fe_2_O_3_ | 0.61 | 6.711×10^9^ | 2.63×10^20^ |
| Co-MOF/Ti:Fe_2_O_3_ | 0.56 | 1.895×10^9^ | 9.32×10^20^ |

**References:**

[1] Wang, Z.-Y., Li, H.-M., Yi, S.-S., You, M.-Z., Jing, H.-J., Yue, X.-Z., et al. (2021). In-Situ Coating of Multifunctional FeCo-Bimetal Organic Framework Nanolayers on Hematite Photoanode for Superior Oxygen Evolution. *Appl*. *Catal*. *B*: *Environ*. 297, 120406. doi: 10.1016/j.apcatb.2021.120406

[2] Wang, H., He, X., Li, W., Chen, H., Fang, W., Tian, P., et al. (2019). Hematite Nanorod Arrays Top-Decorated with an MIL-101 Layer for Photoelectrochemical Water Oxidation. *Chem*. *Commun*. 55, 11382–11385. doi: 10.1039/C9CC05331J

[3] Huang, P., Miao, X., Wu, J., Zhang, P., Zhang, H., Bai, S., et al. (2022). Facile Synthesis of an Ultrathin ZIF-67 Layer on the Surface of Sn/Ti Co-Doped Hematite for Efficient Photoelectrochemical Water Oxidation. *Dalton Trans*. 51, 8848–8854. doi: 10.1039/D2DT00709F

[4] Dong, Y.-J., Liao, J.-F., Kong, Z.-C., Xu, Y.-F., Chen, Z.-J., Chen, H.-Y. et al. (2018). Conformal Coating of Ultrathin Metal-Organic Framework on Semiconductor Electrode for Boosted Photoelectrochemical Water Oxidation. *Appl*. *Catal*. *B*: *Environ*. 237, 9–17. doi: 10.1016/j.apcatb.2018.05.059

[5] Zhang, Q., Wang, H., Dong, Y., Yan, J., Ke, X., Wu, Q., et al. (2018). In Situ Growth of Ultrathin Co-MOF Nanosheets on α-Fe_2_O_3_ Hematite Nanorods for Efficient Photoelectrochemical Water Oxidation. *Sol*. *Energy* 171, 388-396. doi: 10.1016/j.solener.2018.06.086

[6] Wang, K., Liu, Y., Kawashima, K., Yang, X., Yin, X., Zhan, F., et al. (2020). Modulating Charge Transfer Efficiency of Hematite Photoanode with Hybrid Dual-Metal-Organic Frameworks for Boosting Photoelectrochemical Water Oxidation. *Adv*. *Sci*. 7, 2002563. doi: 10.1002/advs.202002563

[7] Cai, J., Tang, X., Zhong, S., Li, Y., Wang, Y., Liao, Z., et al. (2023). Elucidation the Role of Co-MOF on Hematite for Boosting the Photoelectrochemical Performance toward Water Oxidation. *Int*. *J*. *Hydrogen Energy* 48, 12342–12353. doi: 10.1016/j.ijhydene.2022.12.165

[8] Li, L., Zhang, H., Liu, C., Liang, P., Mitsuzaki, N., and Chen, Z. (2019). Effect of Co-MOF Prepared by In-Situ Growth Method on the Photoelectrochemical Performance of Electrodeposited Hematite Photoanode. *Energy Technol*. 7, 1801069. doi: 10.1002/ente.201801069

[9] Liu, X., Zhan, F., Li, D., and Xue, M. (2020). α-Fe_2_O_3_ Nanoarrays Photoanodes Decorated with Ni-MOFs for Enhancing Photoelectrochemical Water Oxidation. *Int*. *J*. *Hydrogen Energy* 45, 28836–28846. doi: 10.1016/j.ijhydene.2020.07.277

[10] Wu, F., Xie, J., You, Y., Zhao, Z., Wang, L., Chen, X., et al. (2020). Cobalt Metal-Organic Framework Ultrathin Cocatalyst Overlayer for Improved Photoelectrochemical Activity of Ti-doped Hematite. *ACS Appl*. *Energy Mater*. 3, 4867–4876. doi: 10.1021/acsaem.0c00465

[11] Xiao, F., Guo, R., He, X., Chen, H., Fang, W., Li, W., et al. (2021). Enhanced Photocurrent by MOFs Layer on Ti-Doped α-Fe_2_O_3_ for PEC Water Oxidation. *Int*. *J*. *Hydrogen energy* 46, 7954–7963. doi: 10.1016/j.ijhydene.2020.12.023

[12] Li, W., Wang, K., Yang, X., Zhan, F., Wang, Y., Liu, M., et al. (2020). Surfactant-Assisted Controlled Synthesis of a Metal-Organic Framework on Fe_2_O_3_ Nanorod for Boosted Photoelectrochemical Water Oxidation. *Chem*. *Eng*. *J*. 379, 122256. doi: 10.1016/j.cej.2019.122256

[13] Liu, G., Zhao, Y., Yao, R., Li, N., Wang, M., Ren, H., et al. (2019). Realizing High Performance Solar Water Oxidation for Ti-Doped Hematite Nanoarrays by Synergistic Decoration with Ultrathin Cobalt-Iron Phosphate Nanolayers. *Chem*. *Eng*. *J*. 355, 49–57. doi: 10.1016/j.cej.2018.08.100

[14] Liu, G., Zhao, Y., Li, N., Yao, R., Wang, M., Wu, Y., et al. (2019). Ti-Doped Hematite Photoanode with Surface Phosphate Ions Functionalization for Synergistic Enhanced Photoelectrochemical Water Oxidation. *Electrochim*. *Acta* 307, 197–205. doi: 10.1016/j.electacta.2019.03.214
